# Superior Mesenteric Artery Dissection after Extracorporeal Shockwave Lithotripsy

**DOI:** 10.1155/2012/168046

**Published:** 2012-12-13

**Authors:** Christos Bakoyiannis, Ioannis Anastasiou, Andreas Koutsoumpelis, Evangelos Fragiadis, Eleni Felesaki, Marina Kafeza, Sotirios Georgopoulos, Christos Tsigris

**Affiliations:** ^1^Vascular Unit, First Department of Surgery, Laiko General Hospital, University of Athens, Athens, Greece; ^2^Department of Urology, Laiko General Hospital, University of Athens, Athens, Greece; ^3^Department of Radiology, Laiko General Hospital, Athens, Greece

## Abstract

The use of shockwave lithotripsy is currently the mainstay of treatment in renal calculosis. Several complications including vessel injuries have been implied to extracorporeal shockwave lithotripsy. We report an isolated dissection of the superior mesenteric artery in a 60-year-old male presenting with abdominal pain which occurred three days after extracorporeal shockwave lithotripsy. The patient was treated conservatively and the abdominal pain subsided 24 hours later. The patient's history, the course of his disease, and the timing may suggest a correlation between the dissection and the ESWL.

## 1. Introduction

The extracorporeal shockwave lithotripsy (ESWL) has become the gold standard in the treatment of renal calculosis especially in the upper lobe. As all therapeutic modalities, ESWL may present with several complications including arterial rupture or dissection regardless of the presence of aortic aneurysm [1–7]. The isolated dissection of the superior mesenteric artery, though, is very rare. We report a case of a 60-year-old male who developed isolated superior mesenteric artery dissection 5 days after ESWL.

## 2. Case Report

**Figure 1:**
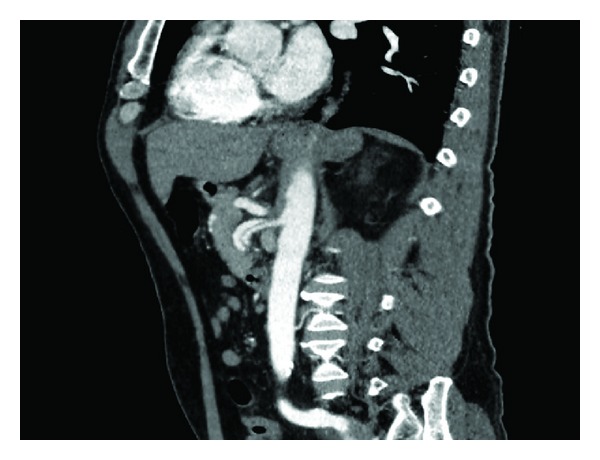
Computed tomography angiogram (sagittal view) depicting the dissection of the superior mesenteric artery extending from the orifice without involving any branch vessel.

**Figure 2:**
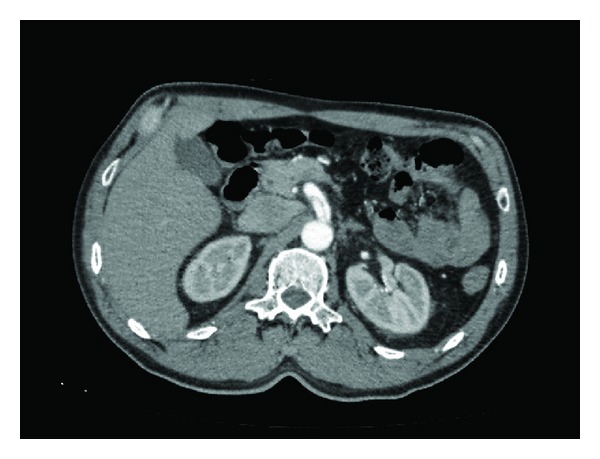
Computed tomography angiogram (axial view) revealing the double-barrel sign of the dissected superior mesenteric artery.

A 60-year-old male, ex-smoker, presented to the emergency department complaining of right colicky pain and high-grade fever 24 hours after being subjected to ESWL. Patient underwent an abdominal ultrasound revealing urolithiasis on the right side along with minimal urine stasis in the right renal pelvis. He was subjected to prophylactic antibiotic coverage and pigtail placement under fluoroscopic guidance with subsequent remission of fever and pain. Forty-eight hours later he complained of acute onset of sharp epigastric pain reflecting to the lumbar region. His physical examination revealed tenderness over the epigastrium with no abdominal palpable mass. The arterial pressure was mildly elevated (140–90 mmHg) and his pulses were 60 bpm. His lab workout revealed a blood count and a basic metabolic panel within normal limits, with the exception of WBC and CRP which were moderately increased. An abdominal duplex ultrasound was performed revealing a flap in the superior mesenteric artery. The CT angiogram of thoracic and abdominal aorta demonstrated an isolated superior mesenteric artery dissection, starting from its orifice, without SMA branch involvement (Figure 1). Both true and false lumens were patent (Figure 2). The diameters of the infrarenal aorta and superior mesenteric artery were normal. Additionally, the aorta and the iliac arteries were found abundant of atheromatous plaques.

The patient was transferred to our vascular department. We decided to start a medical treatment consisting of food and liquid restriction, close surveillance, intravenous administration of beta-blockers, and antithrombotic therapy with unfractionated heparin. After 10 hours the patient's symptoms had diminished and subsided 24 hours later. Liquid restriction was halted on the third day and food restriction on the fourth day of admission to our department. No signs of bowel ischemia were reported during the patient's stay in our department. On the seventh day of admission, the patient underwent a CT angiography revealing no change in the arterial dissection. The patient was discharged ten days after admission and was advised to receive warfarin orally and to undergo a follow-up CT angiography one month later.

## 3. Discussion

The dissection of the superior mesenteric artery is a rare entity usually found in combination with aortic dissection. The isolated superior mesenteric artery dissection is even less frequent. In the current literature 168 cases are reported although according to Foord and Lewis the incidence found in autopsies is 0.06% [2]. Its clinical presentation varies from asymptomatic to life-threatening bowel ischemia or arterial rupture. Predisposing factors in the emergence of this dissection include hypertension, medial degeneration, atherosclerosis, fibromuscular dysplasia, segmental arterial mediolysis, connective tissue disorders, and trauma [8]. This is the first time an isolated dissection after ESWL is reported.

The exact mechanism of vessel injuries after ESWL is still undefined. However, the effects of ultrasound on blood vessels have been studied over the last years. Miller et al. suggested that micro bubbles constrained in blood vessels, after being excited by ultrasound, may damage the endothelium and rupture the vessel wall [3]. This is supported by Chen and his colleagues who showed that bubbles undergo oscillations which deform the vessels on the same scale [4]. The same author performed an experiment on ex vivo rat mesentery, suggesting that the vessel wall disruption after ultrasound is caused by vessel distention or invagination, due to cavitation bubbles [5]. The calcification in the aorta potentiates these effects by acting as an acoustic interface and leads to plaque rupture, especially in areas such as the transitional point from the fixed to mobile part of the SMA.

There are no evidence-based guidelines regarding the use of such therapeutic strategy. Options include the medical approach with or without anticoagulation, open surgery, and endovascular therapy. The decision of therapy was based upon the patient's response to initial treatment, that is, blood pressure control and anticoagulation treatment with unfractionated heparin. Park et al. reported a series of 58 patients with isolated superior mesenteric artery dissection, 53 of them being treated conservatively, 4 surgically, and 1 by SMA stenting [6]. The decision to intervene was based on signs of bowel ischemia and unremitting pain after one week of medical therapy. Most patients presented with acute abdominal pain which subsided after a few days and was attributed to the dissection solely. After 23 months of followup, 43% of patients showed no angiographic change of dissection, 41.3% showed a diminished extent of false lumen, and 15.2% showed a complete remodeling, with most changes occurring in the first 6 months.

Concerning the use of antithrombotic therapy, there is a discrepancy among articles. Several authors support the use of anticoagulation [9], while others consider anticoagulation to oppose the goal of treatment, which is to thrombose and obliterate the false lumen [10]. In this case, we used anticoagulation with heparin intravenously, with subsequent conversion to warfarin orally, as the patient's symptoms subsided the first hours after initial treatment. 

In conclusion, the rarity of this case surely does not support a thorough vascular screening test in patients who will receive shockwave treatment. However, this complication should be kept in mind whenever a patient develops acute abdominal pain after this procedure. The treatment should be optimized according to the patient's clinical status and should be revised in case of symptom aggravation.

## Abbreviations

SME: Superior mesenteric artery
ESWL: Extracorporeal shockwave lithotripsy
WBC: White blood cells CRP, C-reactive protein.
